# Protective effects of autophagy and NFE2L2 on reactive oxygen species-induced pyroptosis of human nucleus pulposus cells

**DOI:** 10.18632/aging.103109

**Published:** 2020-04-22

**Authors:** Zhibiao Bai, Wei Liu, Danshuang He, Yiyang Wang, Weiwei Yi, Changqi Luo, Jieliang Shen, Zhenming Hu

**Affiliations:** 1 Department of Orthopaedic Surgery, The First Affiliated Hospital of Chongqing Medical University, Yuzhong 400016, Chongqing, China; 2 Chongqing Key Laboratory of Molecular Oncology and Epigenetics, The First Affiliated Hospital of Chongqing Medical University, Yuzhong 400016, Chongqing, China

**Keywords:** intervertebral disc degeneration, nucleus pulposus cells, pyroptosis, transcription factor, autophagy

## Abstract

Intervertebral disc degeneration (IDD) is characterized by the decrease of nucleus pulposus cells (NPCs). With the increase of the degree of degeneration, the reactive oxygen species (ROS) in nucleus pulposus tissue increases. Pyroptosis is a newly discovered form of cell death and its relationship with oxidative stress in NPCs remains unclear. This study was performed to investigate the mechanisms of pyroptosis of NPCs under oxidative stress. NPCs were isolated from IDD patients by surgical treatment. Pyroptosis related proteins like NLR family pyrin domain containing 3(NLRP3) and PYD and CARD domain containing (PYCARD) were detected by western blot, and membrane pore formation was observed by hochest33342/PI double staining or scanning electron microscope. The results showed that ROS induced the pyroptosis of NPCs and it depended on the expression of NLRP3 and PYCARD. The increased ROS level also increased transcription factor nuclear factor, erythroid 2 like 2 (NFE2L2, Nrf2) and the autophagy of NPCs, both of which attenuated the pyroptosis. In summary, ROS induces the pyroptosis of NPCs through the NLRP3/ PYCARD pathway, and establishes negative regulation by increasing autophagy and NFE2L2. These findings may provide a better understanding of the mechanism of IDD and potential therapeutic approaches for IDD treatment.

## INTRODUCTION

Intervertebral disc degeneration (IDD) is a common reason of low back pain and has become a huge social and economic burden [1]. The intervertebral disc consists of superior and inferior end plates, jelly-like nucleus pulposus and fibrous annulus [2]. It is generally believed that nucleus pulposus cells (NPCs) are responsible for matrix biosynthesis in the NP region, which first exhibits degenerative changes during disc degeneration [3]. Previous studies have shown that the reduction and dysfunction of NPCs is the main cause of IDD, but its potential mechanism remains to be revealed [4, 5]. Studying the mechanism of NPC reduction will help to find effective targets to restore intervertebral disc function and alleviate the course of IDD.

In addition to the widely recognized apoptosis, cellular reduction can also be caused by other forms of cell death. Pyroptosis is a newly discovered form of cell death and significantly different from apoptosis in terms of cell morphological changes and mechanisms. Apoptosis requires activation of casase-3, -8, -9 and is accompanied by karyopyknosis, lysis of DNA and nucleases, and preservation of plasma membrane integrity. During the process of apoptosis, the contents of apoptotic cells are packaged into apoptotic bodies, which are phagocytosed and cleared by phagocytes without causing inflammation. However, pyroptosis is an inflammatory cell death which can be mediated by activated caspase 1 (CASP1) [6]. As a common activator of CASP1, NLRP3 inflammasome is composed of NLRP3, PYCARD and CASP1 [5]. In the early stage of pyroptosis, small cation-permeable pores are formed on the plasma membrane, which lead to the disappearance of the ion gradient and the osmotic swelling and dissolution of the cell. The most prominent feature of pyroptosis is that it depends on the activation of CASP1 and is accompanied by an increase of inflammatory cytokines of cleaved interleukin (IL)-1β and IL-18 [7]. Our previous studies have revealed that inflammatory factors, especially IL-1β, are closely related to IDD and highly expressed in degenerated intervertebral discs [8]. Since apoptosis is non-inflammatory cell death, it is reasonable to assume that NPCs undergo both apoptosis and pyroptosis during IDD progress. Exploring the pyroptosis of NPCs will help to find new ideas for the treatment of IDD.

In degenerated human intervertebral discs, the production of reactive oxygen species (ROS) increases and is closely related to age-related degeneration including cell senescence and reduction [9, 10]. With the increase of the degree of degeneration, the content of ROS in human intervertebral disc increases while the transcription factor NFE2L2, which increases the expression of antioxidant protein, decreases [11]. It has been previously reported that ROS also activates autophagy in NPCs to prevent cell aging [12]. However, the roles of autophagy induced by ROS, NFE2L2 and ROS in the pyroptosis of NPCs are still unknown. Exploring the role of autophagy and NFE2L2 in the pyroptosis of NPCs will contribute to developing new strategies for the treatment of IDD.

This study aims to reveal the potential mechanism of pyroptosis of NPCs under oxidative stress and the roles of autophagy and the transcription factor NFE2L2 during pyroptosis.

## RESULTS

### Pyroptosis related proteins were increased in NPCs with higher ROS level

**Figure 1:**
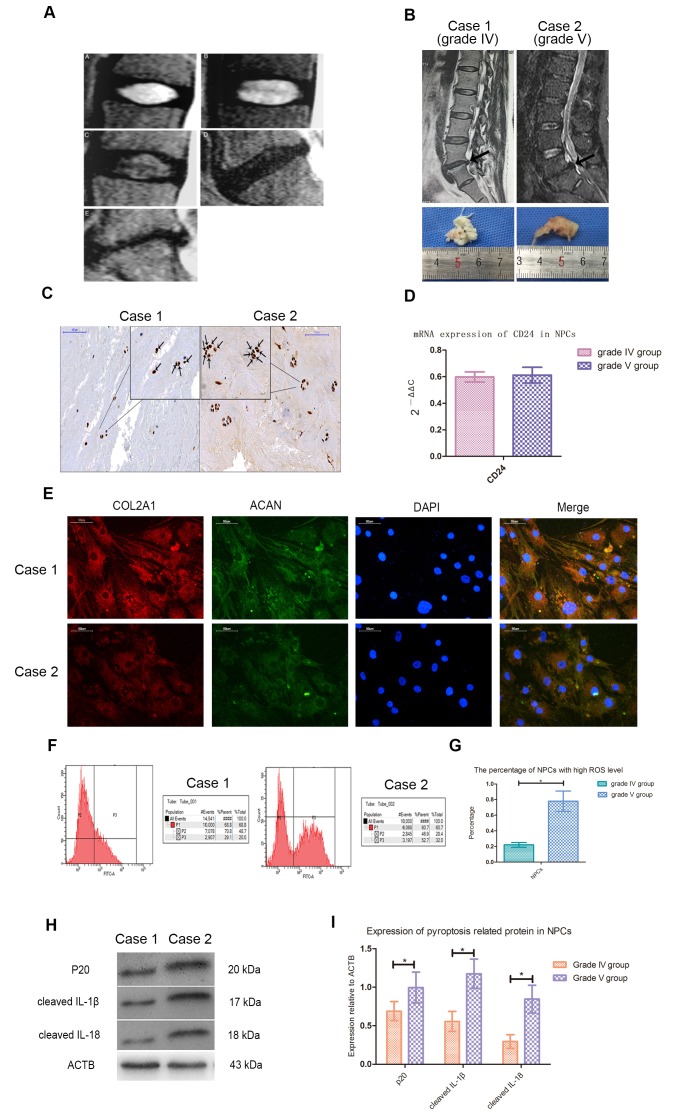
**Cleaved CASP1, IL-1β and IL-18 was higher expressed in NPCs with higher ROS level.** (**A**) Magnetic resonance images of the discs of patients with intervertebral disc degeneration of different Pfirrmann’s classifications. A, B, C, D and E represented the images of patients with grade I, II, III, IV and V intervertebral disc degeneration. (**B**) The representative spinal magnetic resonance images and gross pictures of included patients of grade IV and V treated with transforaminal endoscope. (**C**) The immunohistochemical staining for detection of CASP1 in tissues from patients of grade IV and V (magnification: ×200, scale bar = 100μm). (**D**) The mRNA expression of CD24 in nucleus pulposus cells isolated from included patients with intervertebral disc degeneration of grade IV and V. (**E**) The representative images of immunofluorescence staining for detection of COL2A1 and ACAN in the cultured nucleus pulposus cells from patients of grade IV and V (magnification: ×400, scale bar = 50μm). (**F**) The flow cytometry for detecting the reactive oxygen species level in the cultured nucleus pulposus cells from case1 and case 2. (**G**) The comparison of the percentage of nucleus pulposus cells with high ROS level of patients with intervertebral disc degeneration of grade IV and V. (**H**) The representative western blot images showing the expression of p20, cleaved IL-1β, and cleaved IL-18 in the nucleus pulposus cells isolated from patients of grade IV and V. (**I**) The comparison of pyroptosis related proteins between the patients of grade IV and V based on western blot results. The data were presented as the mean ± SEM. *P < 0.05.

According to Pfirrmann's classification, T2-weighted magnetic resonance imaging (MRI) of intervertebral discs with different degrees of degeneration was shown in Figure 1A. The average age of patients with lumbar disc herniation (LDH) of grade IV and V was 47 and 49 years old, respectively. There was no statistical difference between two groups in age, sex ratio and distribution of surgical stages. Case 1 and case 2 represented LDH patients of grade IV and V, and their imaging and morphological information were shown in Figure 1B. Immunohistochemical staining showed that CASP1 was present in NPCs (Figure 1C). The expression of CD24 in NPCs was identified by qPCR test (Figure 1D), and expression of collagen type II alpha 1 chain (COL2A1) and aggrecan (ACAN) was confirmed by immunofluorescence staining (Figure 1E). Flow cytometry analysis showed that the ROS level of NPCs of patients with grade V was higher than that of patients with grade IV (Figure 1F, 1G). In NPCs from grade V patients, the expression of cleaved IL-1β, IL-18 and CASP1 was also higher (Figure 1H, 1I).

### Hydrogen peroxide induced pyroptosis of NPCs through NLPR3/ PYCARD inflammasome

**Figure 2:**
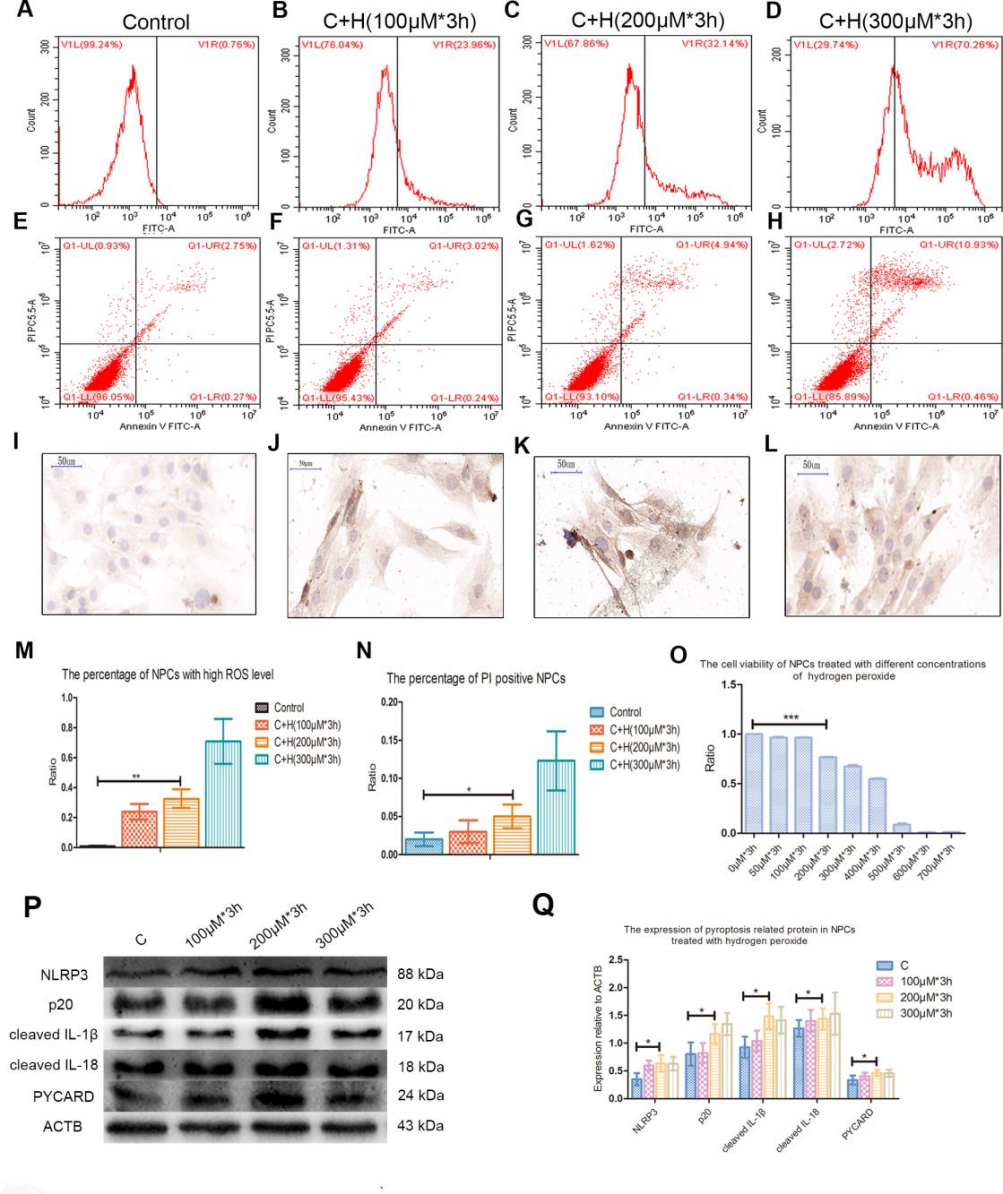
**Hydrogen peroxide induced the pyroptosis of NPCs.** (**A**–**D**) The reactive oxygen species level of the nucleus pulposus cells treated with hydrogen peroxide of 0μM, 100μM, 200μM and 300μM for 3h was detected by flow cytometry. (**E**–**H**) The corresponding apoptosis rates of nucleus pulposus cells treated with different concentrations of hydrogen peroxide were detected by flow cytometry using annexin V/PI double staining. (**I**–**L**) The immunohistochemical staining revealed the expression of CASP1 in the nucleus pulposus cells treated with different concentrations of hydrogen peroxide (magnification: ×40, scale bar = 50μm). (**M**) The panel showed the comparison of percentage of nucleus pulposus cells with high reactive oxygen species level after treatment with hydrogen peroxide of different concentrations. (**N**) The panel showed the percentage of PI positive cells measured after treatment with hydrogen peroxide with different concentrations. (**O**) The CCK-8 test showed the viability of the nucleus pulposus cells treated with different concentration of hydrogen peroxide. (**P**) The expression of NLRP3, cleaved CASP1 (p20), cleaved IL-1β, cleaved IL-18 and PYCARD in the cultured nucleus pulposus cells treated with different concentrations of hydrogen peroxide. (**Q**) The panel showed the averaged data measured from the images as shown in the Figure P. The data were presented as the mean ± SEM. *P < 0.05, **P < 0.01, ***P < 0.001.

**Figure 3:**
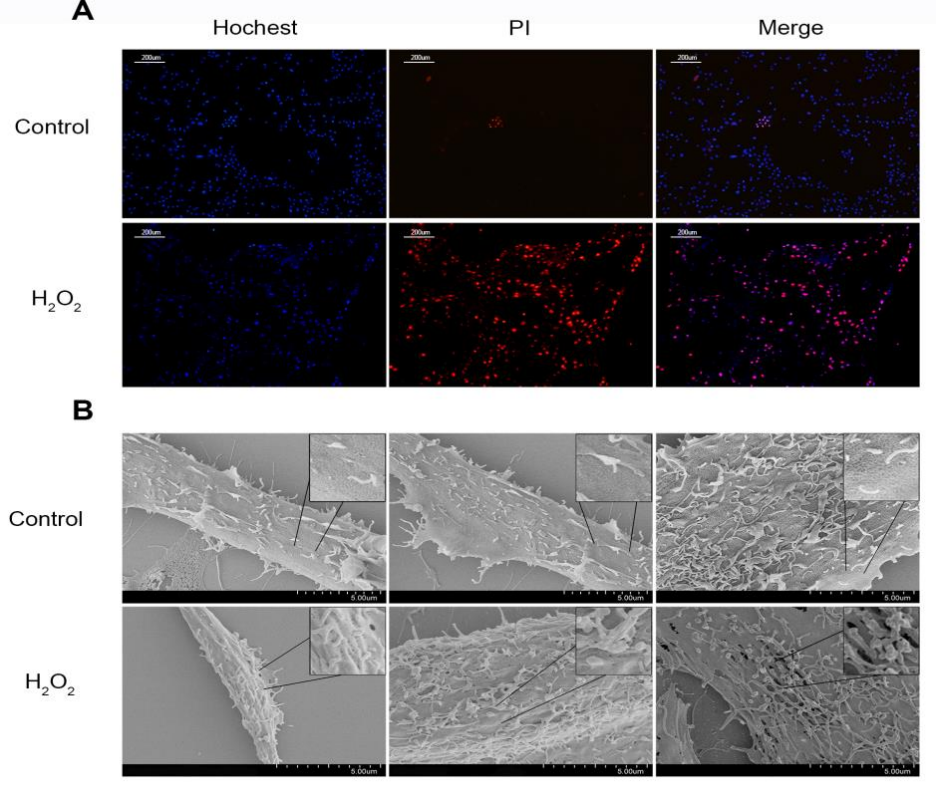
**The change of the cell membrane permeability of NPCs caused by hydrogen peroxide.** (**A**) Hochest33342/PI double staining revealed hydrogen peroxide (200μM, 3h) increased the PI positive nucleus pulposus cells (magnification: ×10, scale bar = 200μm). (**B**) The scanning electron microscopy showed that ball-like bulge and membrane pore-forming were increased by hydrogen peroxide.

Compared with the control group, the ROS level and apoptosis rate of hydrogen peroxide treatment group were increased during flow cytometry test (Figure 2A–2H). Immunohistochemical staining performed on cell climbing slices showed that the positive index of CASP1 expression was also increased in NPCs treated with different concentrations of hydrogen peroxide (Figure 2I–2L). Treatment with 200μmol/L hydrogen peroxide for 3h not only significantly increased the level of ROS and the rate of apoptosis in flow cytometry, but also significantly decreased the viability of NPCs in CCK-8 test (Figure 2M–2O). Western blot analysis showed that the expression of pyroptosis related proteins NLRP3, PYCARD, cleaved CASP1 (p 20), cleaved IL-1β and cleaved IL-18 was increased in NPCs treated with hydrogen peroxide for 3h (Figure 2P, 2Q). Hochest33342/PI double staining showed that PI positive cells were also increased significantly after hydrogen peroxide treatment (Figure 3A). In addition, increased ball-like bulge and membrane pore-forming in hydrogen peroxide treated NPCs were observed by scanning electron microscope (Figure 3B).

### N-Acetyl-L-cysteine (NAC) attenuated NPCs pyroptosis induced by hydrogen peroxide

**Figure 4:**
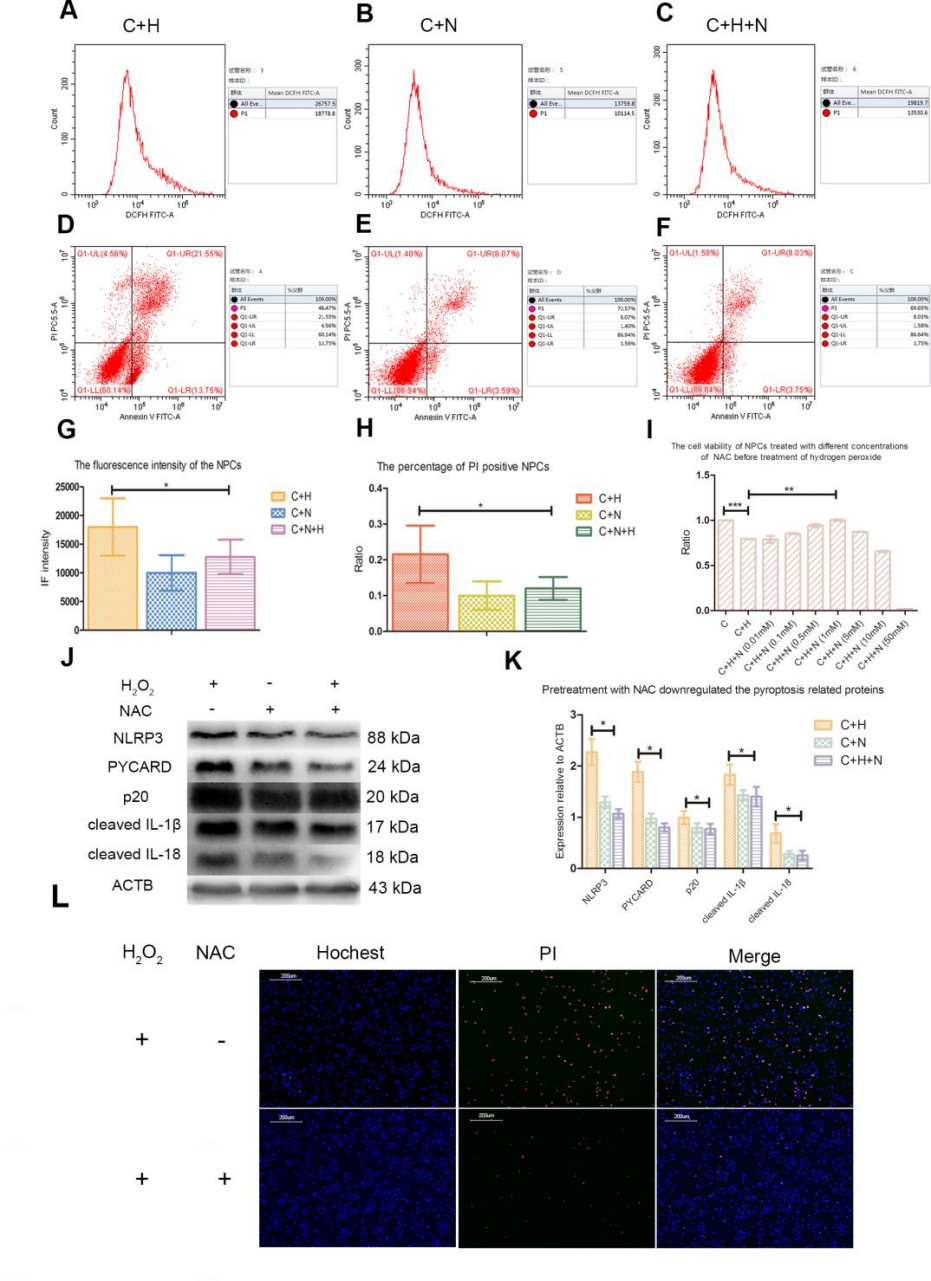
**N-Acetyl-L-cysteine (NAC) attenuated hydrogen peroxide-induced pyroptosis by inhibiting ROS production.** (**A**–**C**) The reactive oxygen species level of the nucleus pulposus cells of C+H, C+N and C+N+H group was detected by flow cytometry. p1 value in the lateral panel revealed the average fluorescence intensity of 1*10^4^ cells. (C+H: The cells treated with hydrogen peroxide; C+N: The cells treated with NAC; C+H+N: The NPCs were pretreated with NAC before treatment with hydrogen peroxide.) (**D**–**F**) The flow cytometer assay showed the rates of PI positive nucleus pulposus cells from the C+H, C+N and C+N+H group in the Q1-UR quadrant. (**G**) The reactive oxygen species levels of the nucleus pulposus cells from C+H, C+N and C+N+H group were compared. (**H**) The rates of PI positive nucleus pulposus cells from C+H, C+N and C+N+H group were compared. (**I**) The CCK-8 test revealed the viability of the nucleus pulposus cells pretreated with different concentrations of NAC before treatment of hydrogen peroxide (200μM, 3h). (**J**) The expression of NLRP3, PYCARD, p20, cleaved IL-1β and cleaved IL-18 in the nucleus pulposus cells of C+H, C+N and C+N+H group was detected by western blot. (**K**) The comparison of the data measured in the Figure J. (**L**) The hochest33342/PI double staining showed that the PI positive cells were decreased by pretreatment of NAC before treatment of hydrogen peroxide (200μM, 3h). (magnification: ×10, scale bar = 200μm) *P < 0.05, **P < 0.01, ***P < 0.001.

Flow cytometry analysis showed that pretreatment with NAC decreased the ROS level and apoptosis rate of NPCs treated with hydrogen peroxide (Figure 4A–4H). CCK-8 analysis showed that NAC with a concentration of 1mmol/L could improve the activity of NPCs treated with hydrogen peroxide (Figure 4I). Pretreatment with NAC also inhibited the upregulation of p20, cleaved IL-1β and cleaved IL-18 in NPCs induced by hydrogen peroxide (Figure 4J, 4K). Fluorescence staining test showed that NAC pretreatment could significantly reduce the proportion of PI positive cells after hydrogen peroxide treatment (Figure 4L).

### Short hairpin RNA targeting NLRP3 or PYCARD (NLRP3-shRNA, PYCARD-shRNA) attenuated the pyroptosis of NPCs induced by ROS

**Figure 5:**
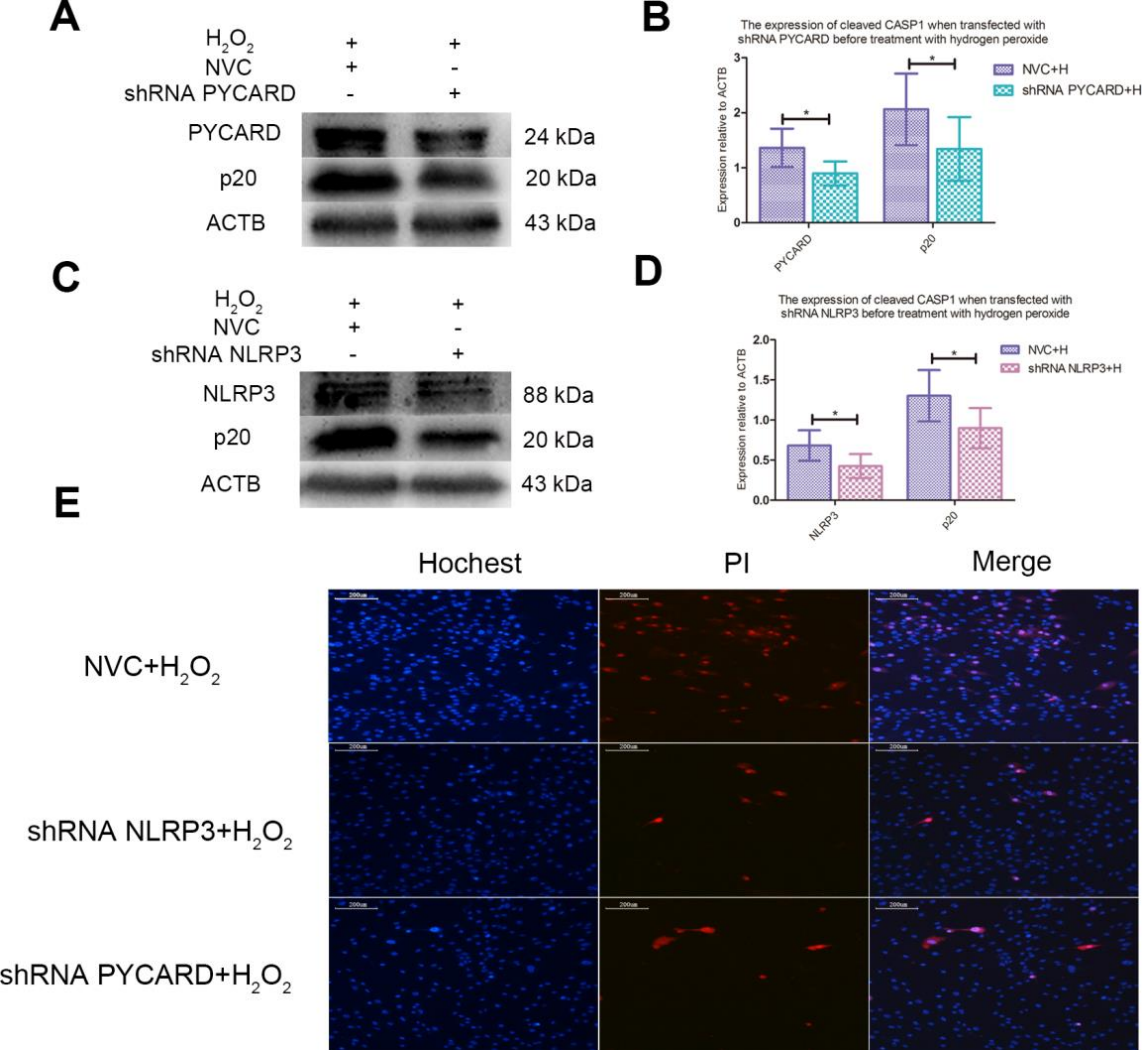
**ROS-induced pyroptosis was NLRP3 and PYCARD dependent.** (**A**) The western blot detecting the expression of cleaved CASP1 and PYCARD in the nucleus pulposus cells transfected with PYCARD-shRNA and non-targeting shRNA (NVC) before treatment with hydrogen peroxide. (**B**) The panel compared the data measured in Figure A. (**C**) The western blot detecting the expression of cleaved CASP1 and NLRP3 in the nucleus pulposus cells after transfection with NLRP3-shRNA and non-targeting shRNA (NVC) before treatment with hydrogen peroxide. (**D**) The panel compared the data measured in Figure C. (**E**) The hochest33342/PI double staining showed that the PI positive cells were decreased when NLRP3 or PYCARD was silenced before treatment with hydrogen peroxide. (magnification: ×10, scale bar = 200μm) The data were represented as mean ± SEM. *P < 0.05.

NLRP3-shRNA and PYCARD-shRNA effectively decreased the expression of NLRP3 and PYCARD, and inhibited the activation of CASP1 induced by ROS in NPCs (Figure 5A–5D). Fluorescence staining test showed that both NLRP3-shRNA and PYCARD-shRNA could reduce the proportion of PI positive NPCs treated with hydrogen peroxide (Figure 5E).

### The autophagy of NPCs was activated to prevent pyroptosis induced by ROS

**Figure 6:**
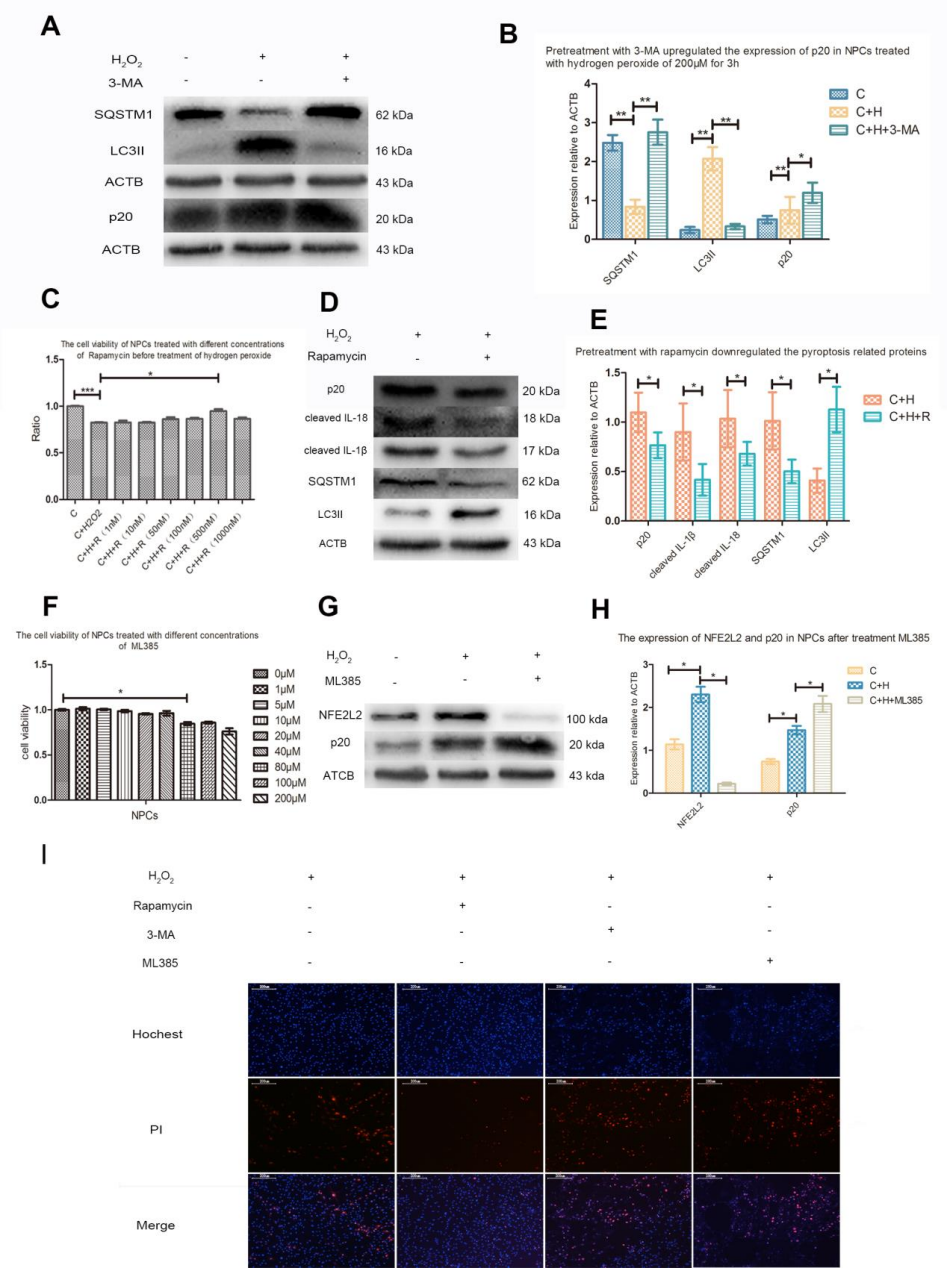
**Autophagy and NFE2L2 both inhibited CASP1 cleavage.** (**A**) The western blot detecting the expression of SQSTM1, MAP1LC3B and p20 in the nucleus pulposus cells with or without pretreatment with 3-MA before treatment with hydrogen peroxide. (**B**) The comparison of the data measured in the Figure A. (**C**) The CCK-8 test revealing the viability of the cells pretreated with different concentration of rapamycin before treatment with hydrogen peroxide (200μM, 3h). (**D**) The western blot detecting the expression of SQSTM1, MAP1LC3B and p20 in the nucleus pulposus cells with or without pretreatment with rapamycin before treatment with hydrogen peroxide. (**E**) The comparison of the data measured in the Figure D. (**F**) The CCK-8 test detecting the effect of ML385 of different concentrations on viability of nucleus pulposus cells. (**G**) The western blot detecting the expression of NFE2L2 and p20 in the NPCs with or without pretreatment with ML385 before treatment with hydrogen peroxide. (**H**) The comparison of the data measured in the Figure G. (**I**) The hochest33342/PI double staining showed the PI positive cells were decreased when nucleus pulposus cells were pretreated with rapamycin and increased when those were pretreated with 3-MA or ML385. (magnification: ×10, scale bar = 200μm) The data were represented as mean ± SEM. *P < 0.05, **P < 0.01.

Pretreatment with 3-MA of 10mmol/L upregulated sequestosome1 (SQSTM1), downregulated microtubule associated protein 1 light chain 3 beta II (MAP1LC3BII, LC3II), promoted the cleavage of CASP1, and increased the PI positives NPCs treated with hydrogen peroxide (Figure 6A, 6B, 6I). CCK-8 analysis showed that rapamycin with a concentration of 500nM increased the activity of NPCs treated with hydrogen peroxide (Figure 6C). Pretreatment with rapamycin also decreased the PI positive NPCs and expression of SQSTM1, cleaved CASP1, IL-1β and IL-18, but increased the expression of LC3II (Figure 6D, 6E, 6I).

### The negative regulation of transcription factor NFE2L2 on ROS-induced pyroptosis

CCK8 test showed that ML385 had no obvious toxic effect on NPCs when the concentration was below 40μM (Figure 6F). Western blot analysis showed that hydrogen peroxide also upregulated the expression of NFE2L2 in NPCs. Compared with NPCs treated with hydrogen peroxide only, the cleavage of CASP1 and PI positive cells were increased in NPCs pretreated with ML385 (Figure 6G–6I).

## DISCUSSION

### The expression characteristics of pyroptosis related proteins in NPCs

After failure of conservative therapy for 3months, IDD patients are recommended for surgical treatment. From our clinical observation, most of the patients with surgical indications are those suffering IDD of grade IV or V according to Pfirrmann's classification. Therefore, the two most common types of patients were included and treated with percutaneous transforaminal endoscopic discectomy (PTED), which made it possible to obtain nucleus pulposus tissue without being contaminated by other cells [13]. According to the results of immunohistochemistry, nucleus pulposus tissue is mainly composed of extracellular matrix such as COL2A1 and ACAN, and only a small part of them is NPCs, which makes it impossible to obtain total cell protein with a high concentration. Therefore, due to objective factors, the tests we can do on the NP tissues were limited, and most of them were carried out in the cultured NPCs. As reported, the cultured cells can be identified as NPCs by detecting the expression of CD24, ACAN and COL2A1 [14].

Unlike apoptosis, pyroptosis is a programmed inflammatory death and characterized by cleavage of CASP1 and release of cleaved IL-1β and IL-18 [7]. The canonical pathway for inducing pyroptosis is CASP1 dependent and can be activated through the danger-associated molecular patterns (DAMPs) and(or) pathogen-associated molecular patterns (PAMPs). But the non-canonical pathway is caspase 4/5 or caspase 11 dependent and generally activated by lipopolysaccharide (LPS) of gram-negative bacteria [15]. Since the intervertebral dis is a bacteria-free environment, in this study, we focused on studying the canonical pathway for inducing the pyroptosis of NPCs. Compared with NPCs from patients with grade IV disc degeneration, the expression of cleaved CASP1, IL-1β and IL-18 was higher in NPCs from patients with grade V disc degeneration. Among the included patients, the ROS level of NPCs was also increased in those with higher degree of disc degeneration, which was consistent with previous report [10]. These evidences were in line with our previous hypothesis that ROS would induce pyroptosis of NPCs. However, the number of patients included in this study was limited and large-scale studies were warranted. To further explore the relationship between ROS and pyroptosis of NPCs, we used hydrogen peroxide to stimulate NPCs from patients with lower degree of degeneration before detecting the pyroptosis related index as Wu described previously [16].

### ROS induced the pyroptosis of NPCs through NLRP3/ PYCARD pathways

The relationship between ROS and CASP1 activation varies with the situation. In vitro experiments of macrophages and monocytes, when the production of ROS is inhibited by compounds or knockdown of NADPH oxidase subunits, the activation of CASP1 is inhibited [17–19]. However, in patients with chronic granulomatosis disease, the impaired ROS production due to genetic defects does not affect or even increases the secretion of IL-1β [20–22]. In the macrophages of superoxide dismutase (SOD)1-deficient mice, higher level of ROS inhibits the activation of CASP1 [23]. In this study, hydrogen peroxide upregulated pyroptosis related proteins of cleaved CASP1, cleaved IL-1β and cleaved IL-18 and increased PI positive NPCs, both of which could be attenuated by pretreatment of NAC. In other words, increased ROS level was responsible for the activation of CASP1 and the pyroptosis of NPCs. After treatment of hydrogen peroxide, the increased and enlarged membrane pores observed by scanning electron microscope also provided powerful evidence for ROS-induced pyroptosis of NPCs. In addition, transfection of NLRP3-shRNA or PYCARD-shRNA inhibited the CASP1 cleavage and decreased the PI positive NPCs treated with hydrogen peroxide, suggesting that NLRP3 and PYCARD were necessary for ROS-induced pyroptosis of NPCs. According to the achieved results, we first reveal that ROS can induce the pyroptosis of NPCs, which depends on the expression of NLRP3 and PYCARD.

### The potential role of autophagy in ROS-induced pyroptosis of NPCs

Autophagy is a conservative cellular behavior that maintains intracellular homeostasis. However, the exact role of autophagy may be reversed under different conditions, and the mechanism of this conditional dependence, from protecting cells to promoting cell death, remains unclear [24]. Our previous studies have revealed that hydrogen peroxide could induce the autophagy of NPCs [12]. However, the role of autophagy in ROS-induced pyroptosis of NPCs remains unclear. To explore the possible relationship between autophagy and ROS-induced pyroptosis of NPCs, we used 3-MA and rapamycin to inhibit and activate the autophagy of NPCs. In line with our hypothesis, ROS-induced pyroptosis of NPCs was aggravated and alleviated when the autophagy was inhibited and activated. We also report for the first time that autophagy is activated during ROS-induced pyroptosis of NPCs and shows negative regulation and self-protection effect. Autophagy is different in different cell lines, therefore, the ultimate effect of elevated ROS level on cellular pyroptosis may be different. This may partly explain the inverse relationship between ROS and CASP1 activation observed in these different situations [17–23].

### Negative regulatory effect of NFE2L2 on ROS-induced pyroptosis of NPCs

The role of NFE2L2 during the inflammasome and CASP1 activation is also controversial. Freigang, S reported that NFE2L2 was essential for cholesterol crystal-induced inflammasome activation and exacerbation of atherosclerosis [25]. Similarly, in THP-1 cells, NFE2L2 is required for inflammasome activation [26–28]. However, in many different inflammatory disease models, the activation of NFE2L2 has been found to be accompanied by the inhibition of NLRP3 inflammasome [29–34]. In NPCs, the relationship between NFE2L2 and pyroptosis is still unclear. It has been reported that NFE2L2 was decreased in the IDD patients with higher degree of degeneration [11]. In this study, cleaved CASP1 was found increased in NPCs of IDD patients with higher degree of degeneration, which suggested a negative correlation between NFE2L2 and pyroptosis of NPCs. To further prove the negative regulatory effect of NFE2L2 on ROS-induced pyroptosis, we used ML385 to inhibit the expression of NFE2L2 and detected the related index after hydrogen peroxide treatment. Compared with NPCs treated with hydrogen peroxide, pretreatment with ML385 downregulated NFE2L2 and increased the CASP1 cleavage and PI positive NPCs, which indicated that NFE2L2 upregulated by hydrogen peroxide also exhibited negative regulatory effect on pyroptosis. Taken together, we have also for the first time revealed that transcription factor NFE2L2 was increased by increasing ROS level and inhibited the pyroptosis of NPCs.

## CONCLUSIONS

**Figure 7:**
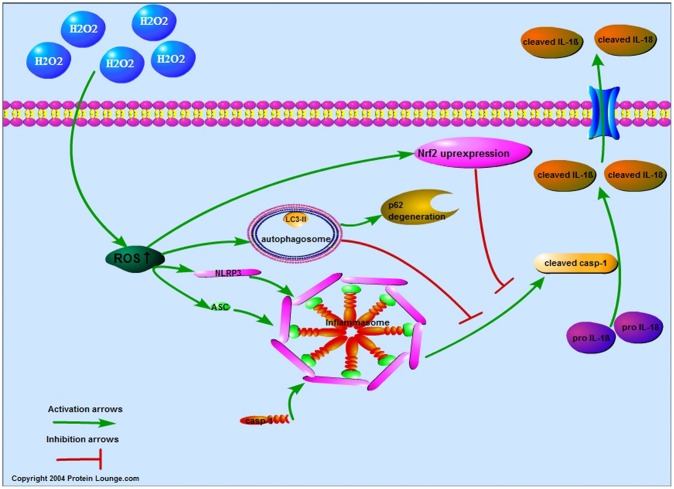
**Proposed model of the ROS-induced pyroptosis and the negative regulation in NPCs.** By increasing the reactive oxygen species level of nucleus pulposus cells, hydrogen peroxide upregulated the expression of NLRP3 and PYCARD to promote the expression of cleaved CASP1, IL-1β and IL-18 and the pore-forming in the membrane. The increased reactive oxygen species also increased the autophagy and NFE2L2 which both attenuated reactive oxygen species induced pyroptosis of nucleus pulposus cells.

In summary, this study evaluated the relationship between oxidative stress and pyroptosis of NPCs. Our results demonstrated that ROS induced the pyroptosis of NPCs through NLRP3/ PYCARD inflammasome and established negative regulation by increasing autophagy and NFE2L2 (Figure 7). This study provides a new idea for studying the decrease of NPCs and a new strategy for the treatment of IDD.

## MATERIALS AND METHODS

### NPC isolation and culture

This study was approved by the ethics committee of the first affiliated hospital of Chongqing medical university. All written informed consents were obtained before surgery. Nucleus pulposus tissue was obtained from patients with LDH treated by PTED. According to Pfirrmann's classification [35], each three patients with IDD of grade IV and V from the age group 35-55 were included to explore the expression characteristics of pyroptosis related proteins. In each group, the herniated lumbar disc was L5/S1. Inclusion criteria: ① Patients without obvious communication obstacles; ② Patients who were diagnosed as LDH according to symptoms, signs and imaging data; ③ Patients who had waist and leg symptoms after failure of conservative therapy for 3 months. Exclusion criteria: ① Patients with other diseases about spine or systemic diseases like diabetes mellitus, hypertension, heart disease, etc. The NPCs isolated from patients with IDD of grade IV were used for further experiment exploring the mechanism of pyroptosis of NPCs. NPCs were isolated and cultured as described previously [36]. Briefly, the nucleus pulposus tissue extracted from the operation was washed with phosphate buffered saline (PBS) containing streptomycin and penicillin, cut by ophthalmology, and digested with 0.25% trypsin and 0.2% type II collagenase (Sigma, USA) at 37°C for 3-5h. The cells in the supernatant were collected by centrifugation and cultured in condition medium consisting of 83% DMEM/F12 medium and 17% fetal bovine serum (Gibico, USA). NPCs were cultured in a humidified incubator with 1% O2, 5% CO2, and 94% N2 at 37°C. The NPCs of second-passage were used for further experiment as described previously [37].

### Cell treatment

According to our previous experience and CCK-8 result, 200μmol/L hydrogen peroxide was added to the medium for 3h to induce the oxidative stress of NPCs. NPCs were pretreated with 1mmol/L of NAC (Sigma, U.S.A, CAS No. :616-91-1) [3] for 1h to exhibit anti-oxidant function, 500nmol/L of rapamycin (Selleck, U.S.A, CAS No. :53123-88-9) [37] and 10mmol/L of 3-MA (Sigma, U.S.A, CAS No. : 5142-23-4) [38] to activate and inhibit the autophagy, and 20μmol/L ML385 (MCE, U.S.A, CAS No. : 846557-71-9) to inhibit the expression of NFE2L2.

### RNA isolation and quantitative real time polymerase chain reaction (RT-qPCR)

The primers of CD24 were designed and synthesized by Shanghai Shenggong Biological Co., Ltd., with actin beta (ACTB) as the internal reference control. (Table 1) According to the manufacturer’s instructions, the total RNA of NPCs was extracted utilizing Trizol reagent (Invitrogen, USA). Then 5μL of RNA was reverse transcribed to achieve cDNA products for amplification. Each group had three duplicates. The obtained Ct value of each group was presented by 2^-ΔΔCt^.

**Table 1:** Primer sequences utilized for RT-qPCR analyses.

**Gene**	**Sequence**
CD24	
Forward	5'-CCCACGCAGATTTATTCCAG-3'
Reverse	5'-GACTTCCAGACGCCATTTG-3'
ACTB	
Forward	5'-GGACTCGTCATACTCCTGCTTG-3'
Reverse	5'-GGAAATCGTGCGTGACATTAAG-3'

### Cell viability assay

Cell counting kit-8(CCK-8) assay was performed to detect the viability of NPCs according to the manufacturer's instructions. Briefly, 1×10^4^ cells/well were inoculated in 96-well plates and incubated with different concentrations of H_2_O_2_ for 3h. After medium change, 100μL basic medium containing 10μL CCK-8 solution was added to each well at 37°C for another 2h. Finally, the absorbance of each well at 450nm was measured by enzyme-labelling measuring instrument (Tecan, Infinite 200 Pro, USA). Each group had three duplicates.

### Reactive oxygen species detection

The intracellular production of ROS was evaluated by DCFH-DA (Beyotime, S0033), which would be oxidized into fluorescent green dichlorofluorescein (DCF) by ROS. Briefly, the treated cells were collected and suspended in diluted DCFH-DA at a concentration of one million to twenty million/mL, and incubated in a 37°C cell incubator for 20mins. Before detection by flow cytometry (BD Biosciences, USA), the cells were washed three times with serum-free cell culture medium to fully remove the DCFH-DA that did not enter the cells.

### Apoptotic incidence detection

The incidence of apoptosis was detected by flow cytometry (BD Biosciences, USA) using annexin V/PI double staining. Briefly, each 1×10^5^ NPCs were collected and incubated with 5μL of annexin V and 5μL of PI at 37°C for 30mins. Then, the samples were analyzed by flow cytometry within 1h.

### Fluorescence staining

Hochest33342/PI double staining was performed to detect the pyroptosis of NPCs as described previously [16]. Briefly, the treated NPCs on 6-well culture-plates were stained with mix solution of hochest33342 and PI at 4°C for 40mins, and then observed under a fluorescence microscope. Normal cells showed low blue/low red light, apoptotic cells showed high blue/low red light, and pyroptosis cells showed low blue/high red light.

### Protein isolation and Western blot analysis

To isolate the total cellular protein, the NPCs were lysed using modified RIPA buffer (Beyotime, China, Cat. No.: P0013B) which was supplemented with 1mmol/L of PMSF on ice following the manufacturer's protocol. Each protein sample (40μg) was resolved by SDS-PAGE (12%) and transferred to PVDF. After transferring, the membrane was blocked with 5% nonfat milk in Tris-buffered saline and tween 20 (TBST) at room temperature for 2h and then incubated overnight with primary anti-NLRP3(1:1000; Abcam, USA, Cat. No.: ab210491), PYCARD (1:500, Santacruz biotechnology, USA, Cat. No.: sc-514414), CASP1 (1:1000, Proteintech, Chicago, USA, Cat. No.: 22915-1-AP), IL-1β (1:500, ABclonal, Boston, USA, Cat. No.: A1112), IL-18(1:500, ABclonal, Boston, USA, Cat. No.: A1115), MAP1LC3B (1:1000, Abcam, USA, Cat. No. ab51520), SQSTM1 (1:1000, Abcam, USA, Cat. No.: ab56416), NFE2L2 (1:1000, Abcam, USA, Cat. No.: ab62352), ACTB (1:500, Santacruz biotechnology, USA, Cat. No.: sc-47778) at 4°C. The membrane was washed with TBST solution for 3 times and incubated with the secondary antibody at room temperature for 1h. The band was visualized using an ECL-Plus detection kit (New Cell and Molecular Biotech Co., Ltd, P10100). The abundance was quantified by densitometry using Quantity One software (Bio-Rad, USA).

### Transfection with adenovirus

For depletion of NLRP3 or PYCARD in NPCs, short hairpin RNA targeting NLRP3 (NLRP3-shRNA 5’ to 3’: GCCAAGAATCCACAGTGTAAC or GCAAAGGGCCATGGACTATTT (Santa Cruz Biotechnology, Dallas, TX, U.S.A.)) or PYCARD (PYCARD-shRNA 5’ to 3’: GGCAATCCCACCAAATCATCC or GCGGAAGCTCTTCAGTTTCAC (Santa Cruz Biotechnology, Dallas, TX, U.S.A.)) was transfected into NPCs using recombinant adenovirus vector (GenePharma, Shanghai, China). Scrambled shRNA with no known mammalian homology (non-targeting shRNA (Santa Cruz Biotechnology, Dallas, TX, U.S.A.)) was used as negative controls. Briefly, NPCs were transfected with NLRP3-shRNA or PYCARD-shRNA with multiplicity of infection (MOI) of 50 for 2h and then cultured in fresh conditional medium for 96h.

### Immunofluorescence staining

The treated cells were fixed with formaldehyde for 10mins and incubated in 1% BSA/10% normal goat serum/0.3M glycine in 0.1% PBS-Tween for 1h to permeabilize the cells and block non-specific protein-protein interactions. The cells were then incubated with the primary anti-COL2A1 (1:50, Santacruz biotechnology, sc-52658) and ACAN (1:200, proteintech, 13880-1-AP) antibody overnight at 4°C. The secondary antibody (green and red) was goat anti rabbit and mouse (proteintech, SA00003-2 and proteintech, SA00009-1) Ig G(H+L) which were used at a dilution of 1 to 50 for 1h. DAPI was used to stain the cell nuclei(blue) and its concentration was 1.43μM. The cells were observed by fluorescence microscope (CTR4000B, Leica). Experiments were repeated three times independently.

### Immunohistochemical staining

The sections of the paraffin-embedded nucleus pulposus tissue were deparaffinized using xylene and rehydrated using decreasing concentrations of ethanol (100, 95, 85, and 75%), followed by immersion in sodium citrate buffer and heating in a steamer for 30mins for antigen retrieval. Then, 3% hydrogen peroxide was used to remove endogenous peroxidase activities, and the sections were blocked with normal goat serum at room temperature for 15mins. The sections were incubated with the primary antibody CASP1 (1:150, Proteintech, Chicago, USA, Cat. No.: 22915-1-AP) overnight at 4°C. The secondary antibody goat anti rabbit IgG-HRP (1:50, Beyotime, China, A0208) was applied at 37°C for 30mins, and the streptavidin-horseradish peroxidase conjugate was added at 37°C for another 30mins. Then, the sections were stained with DAB for 1min and hematoxylin for 10s. Cells were visualized using a microscope (CTR4000B, Leica). Experiments were repeated three times independently.

### Scanning electron microscopic observation

The slides of cells treated with and without hydrogen peroxide were dehydrated by increasing concentrations of ethanol (30%, 40%, 50%, 60%, 70%, 80%, 90% and 100%). After drying in the CO_2_ critical point dryer, the sample was adhered to the sample stage with double-sided conductive tape and sprayed by ion sputter, then observed and photographed by electron microscope (Hitachi, SU8010).

### Data analysis

The data were expressed as mean ± SEM and analyzed by SPSS 22(IBM Corp., USA). The enumeration data like sex ratio were compared with χ2 test. The measurement data were compared with student-t test or one-way ANOVA method. The graphs were produced by GraphPad 5.0 software. A p value less than 0.05 was considered significant.
